# Two-cavities approach for resection of pediatric abdominal neuroblastic tumors: experience of a national reference pediatric onco-surgical center

**DOI:** 10.1007/s00432-022-04027-9

**Published:** 2022-05-06

**Authors:** Cristian Urla, Steven W. Warmann, Andreas Schmidt, Benjamin Mayer, Rupert Handgretinger, Felix Neunhoeffer, Jürgen Schäfer, Jörg Fuchs

**Affiliations:** 1 Department of Pediatric Surgery and Pediatric Urology, University Children’s Hospital of Tuebingen, Hoppe-Seyler-Strasse 3, 72076 Tübingen, Germany; 2 Department of Pediatric Hematology and Oncology, University Children’s Hospital of Tuebingen, Tübingen, Germany; 3 Department of Pediatric Cardiology, Pulmonology and Intensive Care, University Children’s Hospital of Tuebingen, Tübingen, Germany; 4 Department of Diagnostic and Interventional Radiology, University Hospital of Tuebingen, Tübingen, Germany

**Keywords:** Two-cavities approach, Pediatric, Neuroblastoma, Ganglioneuroma, Thoraco- abdominal approach

## Abstract

**Purpose:**

Surgery of complex neuroblastic tumors often requires additional procedures, especially in the situation of tumor extension within thorax and impossibility of securing the aorta above the tumor. These situations prompt the opening of the thoracic cavity. The concern regarding increased operative trauma and morbidity associated with this approach make surgeons reluctant regarding this technique. The aim of this study was to evaluate the efficacy of two-cavities approach based on our experience in a reference pediatric onco-surgical center.

**Methods:**

Between 2003 and 2021, we operated on 232 neuroblastic tumors. 31/232 patients with complex, advanced-stage neuroblastic tumors underwent tumor resection through a two-cavities approach. A retrospective review of patient’s records was performed.

**Results:**

The median age at operation was 48 months (5–180). 23/31 patients presented image-defined risk factors (IDRF). The approach most commonly used was the transverse laparotomy with incision of the diaphragm (*n* = 14), followed by the thoraco-abdominal incision (*n* = 10). Gross total resection (GTR) was achieved in 24 patients, a near-GTR in 4 cases, and an incomplete resection in 3 cases. Median duration of surgery was 288 min (99–900) and median duration of mechanical ventilation was 22 h (0–336). Postoperative complications occurred in 10 patients, 6/10 required surgical reintervention. The 5-year overall survival (OS) was 90% and the 5-year event-free survival (EFS) was 50%.

**Conclusions:**

The two-cavities approach for resection of abdominal neuroblastoma in children is a safe technique with no added morbidity.

## Introduction

Neuroblastoma (NB) is the most common extracranial malignant solid tumor in infants (Fischer et al. 2017; Park et al. 2010). These tumors have a high propensity for encasement of major vessels and infiltration of abdominal organs or delicate anatomic areas such porta hepatis or pancreas (Warmann et al. 2012). As a consequence, there is an ongoing debate regarding the necessity for complete resection in children with high-risk tumors (Warmann et al. 2011). Whereas some authors postulate that incomplete resection is not an adverse factor for patients’ outcome, others reported that children benefit from gross total resection (GTR) in terms of probability of local progression and overall survival (Warmann et al. 2012; Rich et al. 2011; La Quaglia et al. 2004; Fischer et al. 2017; von Allmen et al. 2017).

To achieve a complete resection, an adequate exposure of the tumor and surrounding structures is essential. The complexity is even greater in the upper abdominal region where encasement of the abdominal aorta and its major branches pose a considerable technical challenge (Qureshi and Patil 2012). In these cases, an abdominal approach alone is not sufficient due to the fact that a telescopic effect is encountered when the limits of the surgical field become narrower while proceeding deeper into the dissection (Qureshi and Patil 2012). In these situations, opening of the thoracic cavity is mandatory. However, the concern regarding increased operative trauma and morbidity, post-operative pain and complications that might be associated with a thoraco-abdominal approach is the reason why some surgeons are reluctant with performing this technique. Therefore, there is a lack of literature addressing the two-cavities approach (thoracic and abdominal) for resection of abdominal neuroblastoma in children (Qureshi and Patil 2012).

The aim of the present study was to evaluate the two-cavities approach for resection of abdominal neuroblastoma regarding the extent of resection, incidence of intra-/post-operative complications, and outcome.

## Patients and methods

### Patients

A retrospective review of patient’s records was carried out. The study was approved by the local ethical committee (number 862/2021BO2). Charts of patients undergoing resection of pediatric neuroblastic tumors by a two-cavities approach between March 2003 and September 2021 were analyzed. We assessed patient’s related data, tumor specifications, surgical data, clinical course, and surgical as well as oncological outcome. Data regarding race/ethnicity were not available.

In patients with NB and GNB, preoperative chemotherapy was administered according to the respective protocols of the German Society of Pediatric Oncology and Hematology, of the International Society of Pediatric Oncology or of the Italian Society of Pediatric Hematology and Oncology. One patient with NB underwent upfront resection.

### Diagnostic evaluation

Pretreatment extent of the primary tumors was assessed using high-field MRI or computed tomography (CT). For MRI, the standard sequence protocol contained high resolved T1 and T2-weighted images with fat saturation as well as multiphase contrast enhanced imaging. Additionally, since 2008, diffusion weighted imaging (DWI) was carried out. Since 2013, the tumor volume was assessed with a semi-automated approach in DWI using a dedicated software prototype. Quantitative apparent diffusion coefficient (ADC) values were calculated automatically of the total tumor volume after manual exclusion of necrosis (Gassenmaier et al. 2020).

Multi-detector computed tomography with up to 128 rows was obtained in arterial and portal-venous phase. A tube voltage of 120 kV was used and tube current was adapted to patient’s body weight according to a standard protocol used in our institution.

A collimation of 0.6 mm was chosen for all examinations. Reconstruction of the raw data was performed with 3–5 mm slice thickness, 1 mm high resolution, and 6 mm thin sliding maximum intensity projection. Further coronal and sagittal multi-planar reconstructions with 3 mm slice thickness were obtained. To avoid respiratory artifacts in children below 5 years of age, intubation with mechanical ventilation and hold respiration was carried out. All scans were reviewed in consensus reading by a pediatric radiologist (JS) and a pediatric surgeon (JF and SW). The indication for GTR was made in the national and local multidisciplinary tumor board (MDT) according to the guidelines of the German Society of Pediatric Oncology and Hematology (GPOH).

### Surgical technique

All patients were operated on if partial response (PR) or stable disease (SD) was achieved after induction chemotherapy. Patients with progressive disease (PD) did not undergo surgery.

**Fig. 1:**
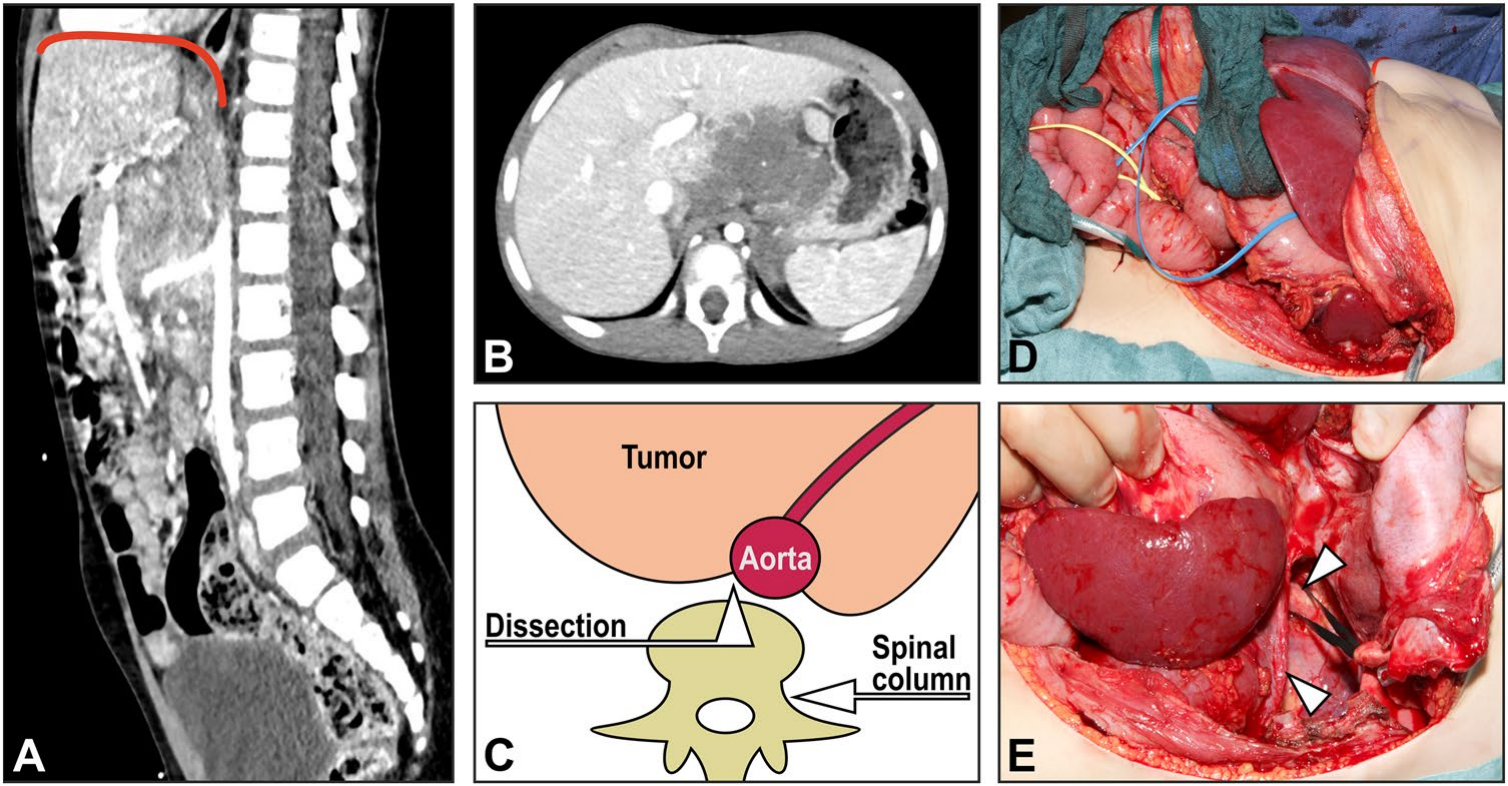
MRI scan images (**A**, **B**) of a patient with abdominal neuroblastoma extending up to the diaphragm (**A**, red curved line); The sketch (**C**) shows the principle of dissection technique, which always begin laterally on a tumor-free part of the aorta; using a thoraco-abdominal approach (**D**), the diaphragm (**E**, lower arrow) was opened and the thoracic aorta (**E**, upper arrow) was secured

The surgical procedures were carried out either by or under assistance of the senior author of this paper (JF). The patients received an epidural catheter for pain management. In the vast majority of the cases, resection of the tumors was carried out either via a transverse laparotomy with subsequent incision of the diaphragm or through a thoraco-abdominal incision. For the latter approach, the transverse laparotomy was extended perpendicular to the thoracic wall in the middle axillary line as shown in Fig. 1. Peripherally, the right or left diaphragm was incised laterally by keeping about 2 cm of the dorsal diaphragmatic margin to avoid injury of the phrenic nerve. The decision on which way the thoracic part of the surgical procedure is performed (thoracotomy, thoracoscopy, and incision of the diaphragm), depends on the individual conditions of each patient or the thoracic tumor localization. After mobilization of the liver, the supra- and infrahepatic inferior vena cava were exposed and encircled by a tourniquet. The aorta was encircled on a vessel loop at the aortic hiatus and superior to the aortic bifurcation. For tumor dissection we always begin laterally on a tumor-free part of the aorta (Fig. 1). The preparation of the tumor from the vessels was performed in the subadventitial plane as previously described by Kiely and Qureshi (Kiely 1993; Qureshi and Patil 2012).

Gross total resection (GTR) was defined as removal of all visible and palpable tumor mases together with regional lymphatics. A near gross total resection (NGTR) was considered if more than 90% of the tumor mass was removed. A macroscopic residual of more than 10% of the tumor mass was defined as incomplete resection (IR).

A chest tube was placed into the pleural cavity of the affected side. Postoperatively, all patients were transferred to the interdisciplinary pediatric intensive care unit.

### Postoperative course and outcome

To assess the post-operative course, we calculated the paediatric multiple organ dysfunction score (P-MODS) and the vasoactive inotropic score (VIS) (Gaies et al. 2010; Graciano et al. 2005). The P-MODS score is an objective measure for assessing organ disfunction, and correlates strongly with mortality (Graciano et al. 2005). Acute kidney injury (AKI) was evaluated by the pediatric Risk-Injury-Failure-Loss-End-Stage Renal Disease (pRIFLE) criteria (Akcan-Arikan et al. 2007). Postoperative complications were classified according to the classification proposed by Dindo and Clavien (Dindo et al. 2004). Descriptive statistics for continuous variables are expressed as median and range. Survival curves were obtained using the Kaplan–Meier estimation.

## Results

Between March 2003 and September 2021, we operated on 232 patients with abdominal neuroblastic tumors. A two-cavities approach was performed in 31 patients. There were 14 females and 17 males with a median age at operation of 48 months (5–180) and a median weight of 13.9 kg (6.6–70). Twenty-three patients had neuroblastoma, five had ganglioneuroblastoma (GNB), and three had ganglioneuroma (GN). From patients with NB/GNB, 14 were assigned to the high-risk group, 13 to the intermediate-risk group, and 1 to the low-risk group. Amplification of N-myc protooncogene could be detected in 6 patients. Patient’s data are shown in Table 1.

**Table 1:** Patient’s data

ID	Age (months)	Sex	Tumor localization	Histology	Grading (Hughes)	INSS	Risk-Group	N-myc	Vascular IDRF	Resection status	Relapse	Outcome
1	48	F	Mid	NB	1A	IV	HR	pos	4	NGTR (95%)	*–*	NED
2	24	F	Adr	NB	3	III	HR	pos	4	IR	PD	DOD
3	5	M	TA	GNB	2	III	IR	neg	2	NGTR (95%)	MR	AED
4	72	M	Adr	GNB	1B	IV	HR	neg	4	NGTR (90%)	PD	AED
5	72	M	TA	NB	1B	IV	HR	neg	1	GTR	PD	NED
6	10	M	Mid	NB	2	IV	IR	neg	6	GTR	*–*	NED
7	48	M	Mid	GNB	1B	IV	HR	neg	4	GTR	*–*	NED
8	24	M	TA	NB	2	III	IR	neg	*–*	GTR	*–*	NED
9	60	M	Mid	GN	*–*	*–*	*–*	neg	7	GTR	*–*	DOC
10	11	F	TA	GNB	1A	IV	HR	neg	2	GTR	*–*	NED
11	60	F	TA	NB	2	IV	HR	neg	6	GTR	CR	AED
12	36	M	TA	NB	3	IV	HR	neg	*–*	GTR	CR	AED
13	24	F	Mid	NB	2	IV	HR	pos	6	GTR	*–*	DOD
14	132	M	Mid	GNB	1A	III	IR	neg	7	GTR	*–*	NED
15	13	M	Mid	NB	2	IV	HR	neg	4	GTR	MR	AED
16	108	M	TA	NB	1A	III	IR	neg	4	GTR	*–*	NED
17	48	M	Mid	NB	1A	III	IR	neg	8	IR	LR	AED
18	96	F	Adr	NB	1A	III	IR	neg	*–*	GTR	*–*	NED
19	18	F	TA	NB	2	III	LR	neg	*–*	GTR	*–*	NED
20	26	M	Mid	NB	2	IV	HR	pos	3	GTR	*–*	NED
21	28	F	Mid	NB	2	IV	HR	neg	6	GTR	*–*	NED
22	20	M	TA	NB	1A	III	IR	neg	*–*	GTR	*–*	NED
23	42	F	Mid	NB	2	III	IR	neg	4	GTR	*–*	NED
24	49	M	Mid	NB	2	IV	IR	neg	3	GTR	*–*	NED
25	56	F	TA	NB	3	IV	HR	pos	2	GTR	*–*	NED
26	59	F	Th	NB	1	III	IR	neg	*–*	NGTR	*–*	NED
27	100	M	Mid	NB	1B	IV	HR	neg	5	GTR	*–*	NED
28	78	F	Mid	NB	3	IV	HR	pos	3	GTR	*–*	NED
29	20	F	TA	NB	1A	III	IR	neg	*–*	GTR	*–*	NED
30	56	M	Mid	GN	*–*	*–*	*–*	*–*	7	IR	*–*	NED
31	180	M	TA	GN	*–*	*–*	*–*	*–*	*–*	GTR	*–*	NED

Histology: *NB* neuroblastoma, *GNB* ganglioneuroblastoma, *GN* ganglioneuroma; INSS: preoperative tumor stages; Risk group: *HR* high risk, *IR* intermediate risk; N-Myc: amplified gene expression of the tumor; Encased vessels: *TC* celiac axis, *AMS* superior mesenteric artery, *AR* renal artery, *VR* renal vein, (b) bilateral, *AMI* inferior mesenteric artery, *VP* portal vein, *VCI* inferior vena cava, *VHD* right hepatic vein; Resection status: *GTR* gross total resection, *NGTR* near gross total resection, *IR* incomplete resection; Relapse: *CR* combined relapse, *MR* metastatic relapse, *PD* progressive disease; Outcome: NED no evidence of disease, *DOD* died of disease, *DOC* died of other cause

**Fig. 2:**
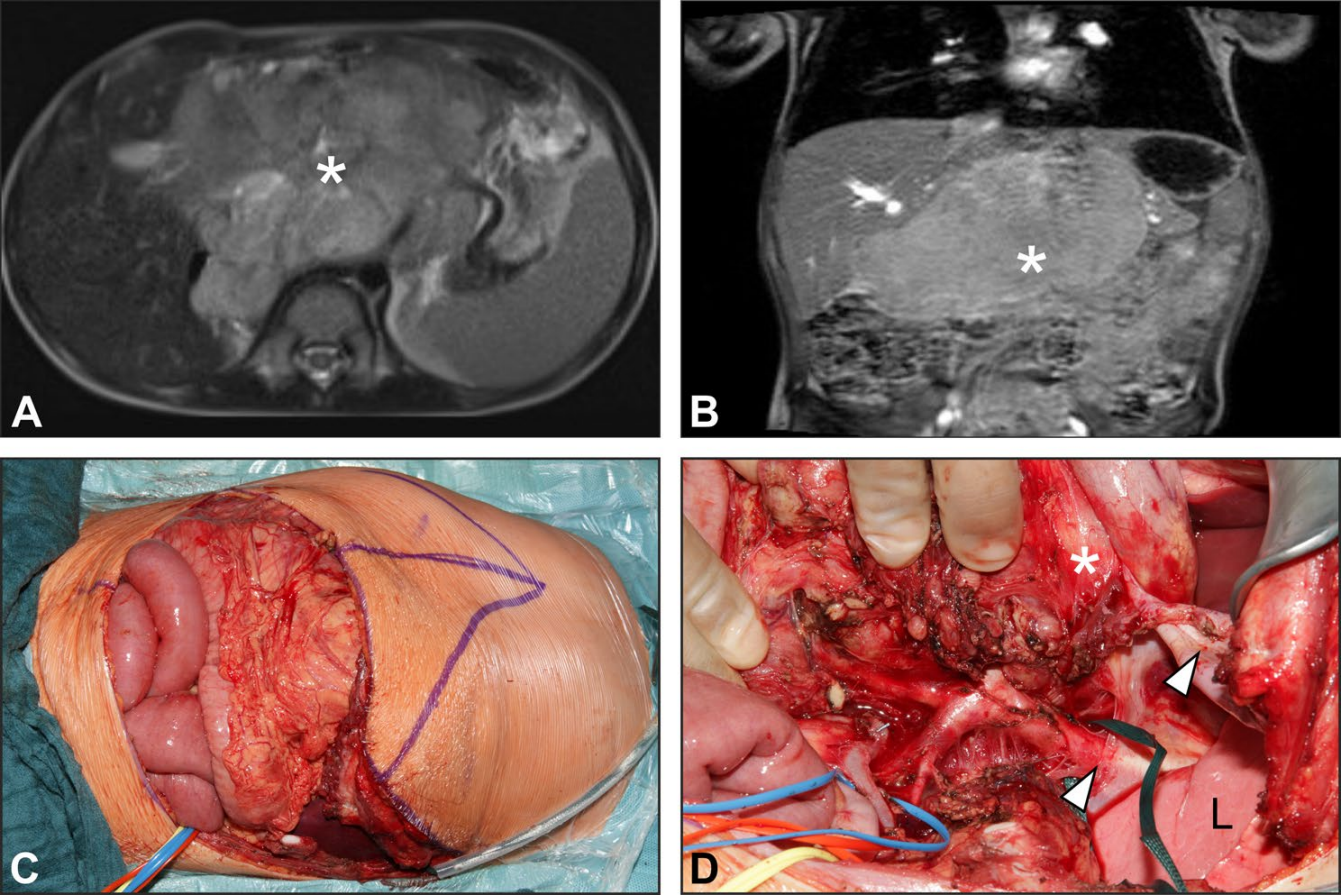
MRI scan of a patient with upper abdominal ganglioneuroma (**A**, **B**) suitable for a thoraco-abdominal approach (**B**), which allowed an adequate exposure of the involved structures (**C**). Lower arrow: abdominal aorta; upper arrow: left hemidiaphragm; L: lung. *Tumor mass

The approach most commonly used was the transverse laparotomy with incision of the diaphragm (*n* = 14), followed by thoraco-abdominal incision (*n* = 10) (Fig. 2), transverse laparotomy with lateral thoracotomy (*n* = 4), laparoscopy with incision of the diaphragm (*n* = 2), and transverse laparotomy with thoracoscopy in one case. In this latter case, the thoracoscopy was performed due to the presence of multiple round foci on both sides of the thorax, and by which no distinction could be made on CT and MRI between aspergilloma and tumor.

Image-defined risk factors (IDRF) were observed in 23 patients. Encasement of more than 3 major vascular structures (celiac axis, superior mesenteric artery, renal artery, inferior mesenteric artery etc.) could be seen in 16/23 cases. Details regarding the number of vascular IDRF are given in Table 1.

To achieve a GTR, 6 patients required extended resections which included other organs (pancreas head, *n* = 1; liver segments VII–VIII and partial nephrectomy, *n* = 1; nephrectomy and hemicolectomy, *n* = 1; nephrectomy, *n* = 1; splenectomy, nephrectomy and pancreas tail, *n* = 1, right hemihepatectomy and pancreas tail resection, *n* = 1). Vascular reconstruction was necessary in three cases. In one patient with NB the reconstruction of the left renal vein was needed. One patient with GN required the reconstruction of the portal vein with GoreTex patch. In another patient with NB, the auto-transplantation of the right kidney was necessary. This latter patient required in the further course a reconstruction of the abdominal aorta with PTFE and Dacron patch due to septic erosion of the celiac axis and superior mesenteric artery. This child was previously treated in another country and underwent a preliminary operation with injury of the abdominal aorta.

Nineteen patients were previously operated in outside institutions. By 9 from these 19 patients, an initial incomplete resection of the tumor was carried out, and the remaining 10/19 patients underwent tumor biopsy.

Gross total resection was achieved in 24 patients, an NGTR (macroscopic residual < 10%) was achieved in 4 cases. Three patients had a gross residual disease after surgery (25% of the initial tumor mass). In one of these three patients, the removal of the tumor from the encased vessels was very difficult due to infiltration of the tumor in the subadventitial plane. Moreover, the pancreas, the spleen, the stomach and the left kidney were infiltrated by the tumor. Taking into consideration the unfavorable constellation (N-myc positive, infiltration of surrounding structures, and progress) after intraoperative discussion with our oncologists the decision was made to remove as much tumor as possible. The remaining two patients had a ganglioneuroma with extensive encasement of the mesenteric vessels. In these cases, the decision to incompletely resect the tumor was made to on avoid injury of the mesenteric vessels.

To avoid the risk of organ perfusion impairment through kinking of the liberated vessels, the abdomen was left open in 8/31 patients. In these cases, the secondary closure of the abdominal wall was carried out after a median of 48 h (72–480).

The median duration of surgery was 288 min (99–900), and the median intraoperative blood loss was 28.3 ml/kg (0–204 ml/kg). Comparing the different histological subtypes, we observed that GN required longer operation time (mean 389.3 ± 120.7 min) compared to NB/GNB patients (mean 309.6 ± 35.8 min).

Details on outcome of the patients during the stay on the pediatric intensive care unit are given in Table 2. Median length of PICU stay was 4.0 days (1–66), median duration of mechanical ventilation was 22 h (0–336). The mean duration of mechanical ventilation was longer in GN patients (54.5 ± 9.5 min) compared to NB/GNB patients (45.3 ± 14.1 min). No reintubation was necessary. Maximal P-MODS and VIS within the first 48 h post-operative was 5.0 (2–18) and 4 (0–63) respectively. All patients developed temporarily AKI, diagnosed according to the pRIFLE criteria; 13 patients met the “risk” criteria, 11 patients met the “injury” criteria, and 4 patients met the “failure” criteria. The pain management consisted of fixed administration of intravenous or oral non-steroidal anti-inflammatory agents and opioids if indicated. In 30 patients an epidural catheter was inserted preoperatively. These children received continuous epidural administration of ropivacaine 0.2% and sufentanyl 0.4 µg/ml. In patients with neuroblastoma, post-operative chemotherapy could be started after a median of 21 days (7–42).

**Table 2:** PICU outcome

Patient characteristics	^a^
Weight (kg)	13.9 [6.6–70]
Duration of surgery (min)	288 [99–900]
Intraoperative red blood cell transfusion (ml//kg)	28.3 [0–204]
Duration of mechanical ventilation (h)	22 [0–336]
Removal chest tube (d)	5 [3–10]
Length of PICU stay (d)	4 [1–66]
Postoperative red blood cell transfusion (ml//kg)	22.5 [8.8–265.4]
Max. lactate (mmol/l)	2.3 [1.4–17]
Max. VIS	4 [0–63]
Max. stage of AKI	no AKI-Risk
Max. P-MODS	5 [2–18]
Discharge hospital (d)	16 [6–90]
Start chemotherapy postoperative (d)	21 [7–42]

*PICU* Pediatric Intensive Care Unit, *AKI* Acute Kidney Injury according to the pRIFLE criteria, *P-MODS* Pediatric Multiple Organ Dysfunction Score, *VIS* Vasoactive-Inotropic Score

^a^All data are expressed as median

Postoperative complications occurred in 10 patients. Details regarding complications and their management are shown in Table 3. Only 6 patients required surgical reintervention under general anesthesia (surgical hemostasis, *n* = 3; removal of an intraabdominal hematoma, *n* = 1; suture of a perforation of the stomach, *n* = 1; reconstruction of abdominal aorta, *n* = 1). One of these 6 patients died 2 weeks after the operation. It is about a 3 years old girl with a midline neuroblastoma, by whom a tumor debulking has been performed in another country and was referred to our institution for completion of tumor resection. A GTR could be achieved, however the patient developed a septic erosion of the abdominal aorta due to a sepsis with Klebsiella pneumoniae. Despite multiple re-interventions the patient could not be saved.

**Table 3:** Postoperative complications and their management

ID	Complication	Grade^a^	Management
1	Pleural effusion	IIIa	Chest tube placement
2	Ascites	IIIa	Drainage
3	Pleural effusion	IIIa	Punction
4	Bleeding	IIIb	Surgical hemostasis
5	***–***	***–***	***–***
6	***–***	***–***	***–***
7	***–***	***–***	***–***
8	***–***	***–***	***–***
9	Bleeding	IIIb	Surgical hemostasis
10	***–***	***–***	***–***
11	Thrombosis celiac axis Intraabdominal hematoma	II IIIb	Anticoagulant therapy Removal of the hematoma
12	***–***	***–***	***–***
13	Septic arrosion of the abdominal aorta	IV	Reconstruction of abdominal aorta
14	***–***	***–***	***–***
15	***–***	***–***	***–***
16	Pleural effusion	IIIa	Chest tube placement
17	***–***	***–***	***–***
18	***–***	***–***	***–***
19	***–***	***–***	***–***
20	Perforation of the stomach	IIIb	Suture of the perforation
21	***–***	***–***	***–***
22	***–***	***–***	***–***
23	***–***	***–***	***–***
24	***–***	***–***	***–***
25	***–***	***–***	***–***
26	***–***	***–***	***–***
27	***–***	***–***	***–***
28	***–***	***–***	***–***
29	***–***	***–***	***–***
30	Bleeding	IIIb	Surgical hemostasis
31	***–***	***–***	***–***

^a^According to the classification proposed by Dindo and Clavien

Interestingly, a 5 year-old girl developed postoperatively an acute cardiac failure with dilated cardiomyopathy and ischemic myocardial signs. The Takotsubo-like cardiomyopathy was reversible after symptomatic treatment and cardiac dysfunction completely recovered.

No diaphragmatic paralysis associated with incision of the diaphragm occurred in any patients.

### Outcome

**Fig. 3:**
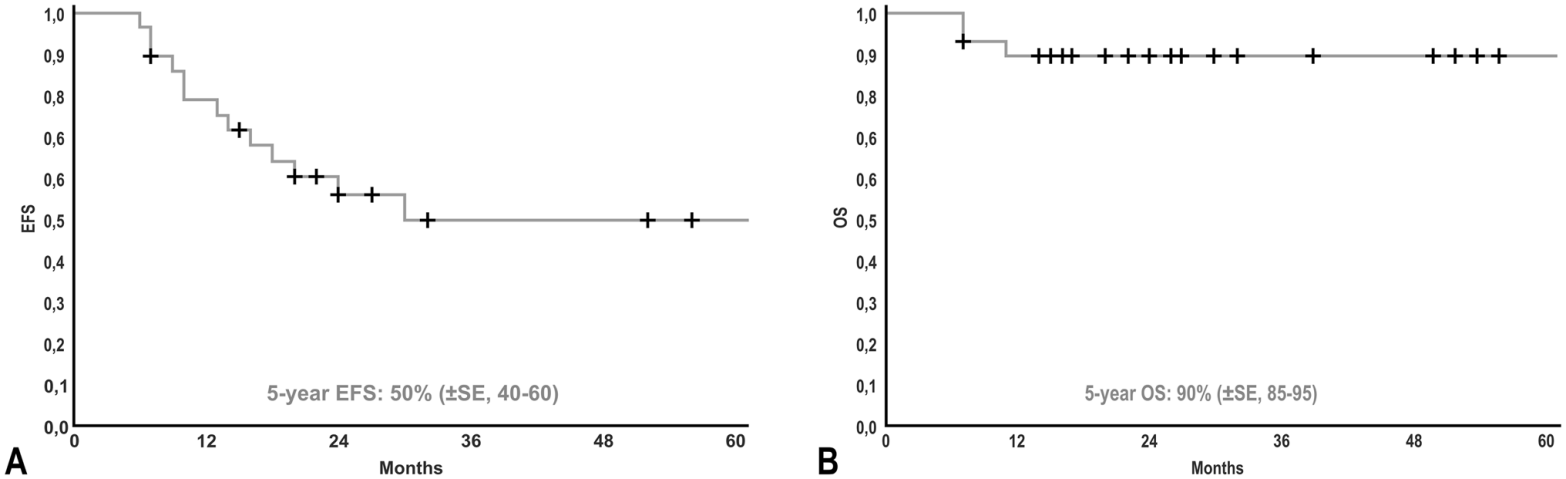
Kaplan–Meier estimation showing 5-year EFS (**A**) and 5-year OS (**B**) in patients who underwent resection of abdominal neuroblastic tumors through a two-cavities approach

Twenty-eight patients are currently alive and two died of disease after a median follow-up of 30 months (7–263). The patient with GN, who required the reconstruction of the portal vein with GoreTex patch, developed a stricture of the portal vein. He died after interventional percutaneous transhepatic portal vein dilatation with subsequent gastroduodenoscopy due to the rare complication of pulmonary embolism with retrograde cerebral venous embolization. Figure 3 shows the Kaplan–Meier survival curves with a 5-year overall survival of 90% (85–95, ± SE, standard error). The 5-year event-free survival was 50% (40–60, ± SE). No surgery-related death occurred.

Tumor relapse occurred in 5 patients (combined relapse (CR), *n* = 2; metastatic relapse (MR), *n* = 2; local relapse (LR), *n* = 1), while 3 patients presented progressive disease (PD). Regarding resection status, 2 patients had an incomplete resection (PD *n* = 1, LR *n* = 1), 2 had near-GTR (MR *n* = 1, PD *n* = 1) and the remaining 4 patients had a GTR (CR *n* = 2, MR *n* = 1, PD *n* = 1).

None of the patients with tumor relapse died. They are still alive but not free of tumor. Only one patient with PD died. The other two patients are still alive and received palliative radiotherapy of the affected regions.

## Discussion

There has been a controversy for some time regarding the benefit of GTR in certain conditions of neuroblastoma. Earlier studies implied a missing relevance of the extent of surgical resection for the outcome of affected patients (Simon et al. 2017; McGregor et al. 2005; von Schweinitz et al. 2002). However, more recent studies produced a trend in favor of GTR as surgical concept in numerous conditions. Fischer et al. reported that in patients with localized neuroblastoma, age 18 months or older, especially in INRG high-risk patients with MYCN amplification, extended surgery of the primary tumor site improved local control rate and survival with an acceptable risk of complications (Fischer et al. 2017). Holmes et al. observed in a recent SIOPEN study that in patients with stage 4 high-risk neuroblastoma who have responded to induction chemotherapy, complete macroscopic excision of the primary tumor was associated with improved survival and local control after high-dose therapy, local radiotherapy (21 Gy), and immunotherapy (Holmes et al. 2020). Liu et al. described in a COG study that boost radiotherapy to gross residual tumor present at the end of induction did not significantly improve the 5-year cumulative incidence of local progression suggesting that there is a need for other local treatment concepts than incomplete resection and boost irradiation (Liu et al. 2020).

Indication for surgery, and implicit for complete tumor resection, in all our patients is established and confirmed by a multidisciplinary tumor board (MDT), locally and in some cases also on a national level. Nevertheless, GTR should be performed with highest safety standards for the patients. This includes preoperative optimization of the patients’ conditions, and the meticulous diagnostic workup regarding tumors and all anatomical structures, as well as during surgery the securing of all vital vessels for an optimal bleeding management. This is especially important regarding the technique of tumor resection in affected patients where dissection should start in the periphery of the tumors after distally securing the vital vascular structures. Here, the two-cavity approach offers an important option. In some cases, there is not enough space to secure the aorta cranially due to the tumor extension toward the diaphragm. In such conditions a trans-diaphragmatic approach allows an improved exposure to the surgeon as well as a safe access to securing the aorta in its thoracic course.

Surgical complexity represents a relevant aspect in the decision-making process regarding the achievement of GTR in patients with advanced NB. The recently proposed surgical complexity index for neuroblastoma is addressing this issue (Loukogeorgakis et al. 2021). Applying this index to the patient cohort analyzed in this study, our patients proved to be extraordinarily complex, especially because of the number of IDRF, which regularly was more than 3. Also, previous operations and previous irradiation treatments often raised the surgical complexity index in our patients, as did a high amount of organ infiltration through vital tumor compounds (for example in the liver), multiple tumor localizations, and intensified preoperative chemotherapy courses. We observed in our patient cohort a large number of intra- and post-operative complications arising from the cited tumor complexity. Vascular injuries and impaired organ perfusion have to be taken into consideration in this regard, a fact, that has also been described by other authors as well (McGregor et al. 2005). Qureshi et al. reported on an incidence of post-operative complications (chyle ascites, small bowel obstruction, and wound infection) in about 37% of the patients in his analysis of the thoraco-abdominal approach for resection of upper abdominal neuroblastoma (Qureshi and Patil 2012). In this study, the authors stated that the complications were rather related to the extensive nature of surgery than to the surgical approach itself. Therefore, with the aim of increasing patients’ safety during surgery in the best way possible, the two-cavity-approach represents a valid option for surgical access.

The course of intensive care unit was generally low in complications. Median duration of post-operative mechanical ventilation was less than 24 h, and median length of PICU stay was 4 days. The mortality rate in the intensive care unit was low at 3% and thus lower than expected by the P-MODS. One death occurred due to septic erosion of the abdominal aorta with Klebsiella pneumoniae. Although all patients met pRIFLE criteria only 4 patients met the “failure” criteria, and no patient met the “loss” criteria.

One of the most challenging groups of tumors regarding GTR in our view is the group of ganglioneuroma. This group contains tumors with the highest numbers of IDRF; also, duration of surgery was the longest in affected children. Ganglionuroma sometimes show an invasive and infiltrating growth pattern concerning vessels and organs. Often, these tumors are large (without option of achieving a preoperative shrinkage through chemotherapy) and affect a large portion of the vascular midline axis. Especially when the celiac trunk or the superior mesenteric artery is elongated and thinned because of their course through the encasing tumor, there is a relevant risk of organ perfusion impairment when the liberated vessels shrink together and show a kinking phenomenon after tumor resection. To prevent a further damaging effect of this, we tend to accept for some days an open abdomen after surgery in certain cases with a prolonged immediate post-operative course and therefore an optimization of the patient’s conditions on the Intensive Care Unit. Usually this is possible and does not mean a burden for the patients from an oncological standpoint because children with ganglioneuroma do not need to be scheduled for a post-operative chemotherapy, which in some instances is crucial to be performed rapidly following the operation.

In conclusion, the two-cavity approach is associated with an improved exposure for resection of large and/or complex abdominal neuroblastoma compared to the pure abdominal access. It facilitates securing of the vital vascular structures at the beginning and during dissection of the tumors and thus contributes to a high level of safety during operation. Finally, this approach is tolerated well by the children and does not lead to increased rates of morbidity, and does not delay the initiation of further scheduled treatment components.

## References

[CR1] Akcan-Arikan A, Zappitelli M, Loftis LL, Washburn KK, Jefferson LS, Goldstein SL (2007). Modified RIFLE criteria in critically ill children with acute kidney injury. Kidney Int.

[CR2] Dindo D, Demartines N, Clavien PA (2004). Classification of surgical complications: a new proposal with evaluation in a cohort of 6336 patients and results of a survey. Ann Surg.

[CR3] Fischer J, Pohl A, Volland R, Hero B, Dubbers M, Cernaianu G, Berthold F, von Schweinitz D, Simon T (2017). Complete surgical resection improves outcome in INRG high-risk patients with localized neuroblastoma older than 18 months. BMC Cancer.

[CR4] Gaies MG, Gurney JG, Yen AH, Napoli ML, Gajarski RJ, Ohye RG, Charpie JR, Hirsch JC (2010). Vasoactive-inotropic score as a predictor of morbidity and mortality in infants after cardiopulmonary bypass. Pediatr Crit Care Med.

[CR5] Gassenmaier S, Tsiflikas I, Fuchs J, Grimm R, Urla C, Esser M, Maennlin S, Ebinger M, Warmann SW, Schäfer JF (2020). Feasibility and possible value of quantitative semi-automated diffusion weighted imaging volumetry of neuroblastic tumors. Cancer Imaging.

[CR6] Graciano AL, Balko JA, Rahn DS, Ahmad N, Giroir BP (2005). The Pediatric Multiple Organ Dysfunction Score (P-MODS): development and validation of an objective scale to measure the severity of multiple organ dysfunction in critically ill children. Crit Care Med.

[CR7] Holmes K, Pötschger U, Pearson ADJ, Sarnacki S, Cecchetto G, Gomez-Chacon J, Squire R, Freud E, Bysiek A, Matthyssens LE, Metzelder M, Monclair T, Stenman J, Rygl M, Rasmussen L, Joseph J-M, Irtan S, Avanzini S, Godzinski J, Björnland K, Elliott M, Luksch R, Castel V, Ash S, Balwierz W, Laureys G, Ruud E, Papadakis V, Malis J, Owens C, Schroeder H, Beck-Popovic M, Trahair T, de Lacerda AF, Ambros PF, Gaze MN, McHugh K, Valteau-Couanet D, Ladenstein RL, For the International Society of Paediatric Oncology Europe Neuroblastoma Group (2020). Influence of Surgical Excision on the Survival of Patients With Stage 4 High-Risk Neuroblastoma: A Report From the HR-NBL1/SIOPEN Study. J Clin Oncol.

[CR8] Kiely EM (1993). Radical surgery for abdominal neuroblastoma. Semin Surg Oncol.

[CR9] La Quaglia MP, Kushner BH, Su W, Heller G, Kramer K, Abramson S, Rosen N, Wolden S, Cheung NK (2004). The impact of gross total resection on local control and survival in high-risk neuroblastoma. J Pediatr Surg.

[CR10] Liu KX, Naranjo A, Zhang FF, DuBois SG, Braunstein SE, Voss SD, Khanna G, London WB, Doski JJ, Geiger JD, Kreissman SG, Grupp SA, Diller LR, Park JR, Haas-Kogan DA (2020). Prospective evaluation of radiation dose escalation in patients with high-risk neuroblastoma and gross residual disease after surgery: a Report from the Children's Oncology Group ANBL0532 Study. J Clin Oncol.

[CR11] Loukogeorgakis S, Sivaraj J, Jain N, Duncan C, Chowdhury T, Giuliani S, Anderson J, Barone G, Cross K (2021). Delayed surgery in high-risk neuroblastoma to prevent intraoperative renal injury. Pediatr Crit Care Med.

[CR12] McGregor LM, Rao BN, Davidoff AM, Billups CA, Hongeng S, Santana VM, Hill DA, Fuller C, Furman WL (2005). The impact of early resection of primary neuroblastoma on the survival of children older than 1 year of age with stage 4 disease: the St. Jude Children’s Research Hospital Experience. Cancer.

[CR13] Park JR, Eggert A, Caron H (2010). Neuroblastoma: biology, prognosis, and treatment. Hematol Oncol Clin N Am.

[CR14] Qureshi SS, Patil VP (2012). Feasibility and safety of thoracoabdominal approach in children for resection of upper abdominal neuroblastoma. J Pediatr Surg.

[CR15] Rich BS, McEvoy MP, Kelly NE, Oh E, Abramson SJ, Price AP, Cheung NK, La Quaglia MP (2011). Resectability and operative morbidity after chemotherapy in neuroblastoma patients with encasement of major visceral arteries. J Pediatr Surg.

[CR16] Simon T, Hero B, Schulte JH, Deubzer H, Hundsdoerfer P, von Schweinitz D, Fuchs J, Schmidt M, Prasad V, Krug B, Timmermann B, Leuschner I, Fischer M, Langer T, Astrahantseff K, Berthold F, Lode H, Eggert A (2017). 2017 GPOH guidelines for diagnosis and treatment of patients with neuroblastic tumors. Klin Padiatr.

[CR17] von Allmen D, Davidoff AM, London WB, Van Ryn C, Haas-Kogan DA, Kreissman SG, Khanna G, Rosen N, Park JR, La Quaglia MP (2017). Impact of extent of resection on local control and survival in patients from the COG A3973 study with high-risk neuroblastoma. J Clin Oncol.

[CR18] von Schweinitz D, Hero B, Berthold F (2002). The impact of surgical radicality on outcome in childhood neuroblastoma. Eur J Pediatr Surg.

[CR19] Warmann SW, Seitz G, Schaefer JF, Scheel-Walter HG, Leuschner I, Fuchs J (2011). Vascular encasement as element of risk stratification in abdominal neuroblastoma. Surg Oncol.

[CR20] Warmann SW, Seitz G, Fuchs J (2012). Surgical complications in pediatric surgical oncology. Pediatr Blood Cancer.

